# The Motor-sparing Paradigm in Knee Analgesia: Advancing Multi-block Strategies with the BiFeS Block as A New Player

**DOI:** 10.4274/TJAR.2026.252351

**Published:** 2026-04-15

**Authors:** Tayfun Et, İlke Dolğun, Muhammet Korkusuz, İbrahim Tülüce

**Affiliations:** 1 Karamanoğlu Mehmetbey University Faculty of Medicine, Department of Anaesthesiology and Intensive Care Medicine, Karaman, Türkiye; 2 University of Health Sciences Türkiye, İstanbul Haseki Training and Research Hospital, Clinic of Anaesthesiology and Reanimation, İstanbul, Türkiye; 3 Karamanoğlu Mehmetbey University Faculty of Medicine, Department of Orthopedics and Traumatology, Karaman, Türkiye

**Keywords:** Adductor canal block, biceps femoris short head block, motor-sparing, orthopaedic anaesthesia, regional anaesthesia

Dear Editor,

Advances in anatomical understanding and enhanced recovery pathways have reshaped regional anaesthesia strategies for total knee arthroplasty (TKA). Motor-sparing techniques have gained increasing importance, reflecting modern priorities such as early mobilisation, reduced fall risk, and optimised functional recovery. This evolution extends beyond the practice patterns described in the review published in the Turkish Journal of Anaesthesiology and Reanimation.^1^ Recent systematic evaluations have further highlighted the growing role of selective, function-preserving regional techniques in contemporary perioperative care.^2^

Despite progress with anterior-based approaches such as the adductor canal block (ACB), posterolateral knee pain remains a frequently under-addressed component of postoperative discomfort. Anatomical studies have clarified the sensory innervation of the posterior knee capsule,^3^ demonstrating contributions from superolateral genicular branches that are not consistently covered by traditional anterior or periarticular techniques. This understanding has stimulated the development of more selective posterior interventions aligned with current motor-sparing goals.

Among these innovations, the biceps femoris short head (BiFeS) block represents a recent anatomically targeted addition. First described by Kilicaslan et al.,^4^ the BiFeS block was designed to target the articular branches that innervate the posterolateral aspect of the knee joint by promoting local anaesthetic spread along the facies poplitea toward the superior lateral genicular nerve and adjacent articular branches, while largely sparing the main motor nerves.^4, 5^ This anatomical selectivity has been proposed as an explanation for its posterolateral analgesic effect without clinically relevant motor impairment.

The combined use of ACB and BiFeS blocks has recently been incorporated into our clinical practice. We present a representative case to illustrate the technique and its potential functional profile.

A sixty-two-year-old male patient (American Society of Anesthesiologists physical status II) underwent unilateral TKA. Informed consent was obtained from the patient for both the procedure and the publication of this case. With the patient in the supine position, baseline isometric strength of the foot muscles was assessed using a handheld dynamometer. Plantar flexion measured 13.1 kilogram-force (kgf) and dorsiflexion measured 9.4 kgf. After mild sedation (midazolam 1 mg, fentanyl 25 μg, and dexamethasone 8 mg , administered intravenously), an ACB was performed with 15 mL of 0.25% bupivacaine.

Ultrasound examination for the BiFeS block was performed using a high-frequency linear transducer (5-18 MHz), with the patient supine and a bolster placed under the leg to be blocked, facilitating access to the posterolateral thigh (Figure 1a). The probe was initially placed in a posterolateral orientation, approximately 20 cm proximal to the lateral femoral epicondyle. It was then slid distally until the short head of the biceps femoris appeared as a narrow, elongated structure. The probe was further moved distally, where the muscle assumed a more quadrilateral configuration, allowing differentiation from the long head of the biceps femoris (Figure 1b). The needle insertion point was selected just distal to the attachment of the adductor magnus to the linea aspera, approximately 8-10 cm proximal to the tip of the lateral femoral epicondyle along the lateral supracondylar line of the femur. Using an in-plane lateral-to-medial approach, the needle was advanced to the interface between the femur and the BiFeS muscle. A total of 20 mL of 0.25% bupivacaine was injected. Correct needle placement was confirmed by observing the linear spread of the local anaesthetic along the femoral surface and the elevation of the muscle (Figure 1c).

One hour after the block, repeat dynamometry showed minimal change in strength (plantar flexion: 12.8 kgf; dorsiflexion: 9.3 kgf). Spinal anaesthesia was then administered in the sitting position using 2.5 mL of 0.5% hyperbaric bupivacaine without intrathecal opioids.

Postoperative multimodal analgesia consisted of paracetamol (1 g three times daily) and dexketoprofen (50 mg twice daily), with oral oxycodone (5 mg) reserved for numerical rating scale pain score ≥4. Resting pain scores at 2, 4, 6, 8, 12, and 24 hours postoperatively were 1, 2, 2, 2, 4, and 4, respectively. No opioids were required during the first 8 hours, and total oxycodone consumption over 24 hours was 15 mg. The modified Bromage score was 4 at 5 hours postoperatively, and the patient was mobilised at 8 hours postoperatively.

In this single case, we observed a reduction in posterolateral knee pain, preservation of quadriceps and peroneal motor function, and early mobilisation. These preliminary findings suggest that the combined ACB and BiFeS approach may be feasible and function-preserving when performed in the supine position; however, further systematic investigation is required before drawing conclusions regarding reproducibility, comparative effectiveness, or routine clinical applicability.

Overall, developments in knee analgesia increasingly reflect a shift toward selective, anatomy-guided, motor-sparing strategies. Within this evolving landscape, particularly in the context of multimodal peripheral block combinations, the BiFeS block represents a potentially useful addition to address the persistent posterolateral analgesic gap. Its integration with established anterior approaches, such as the ACB, may offer a rational pathway for exploration in future clinical studies.

## Figures and Tables

**Figure 1:**
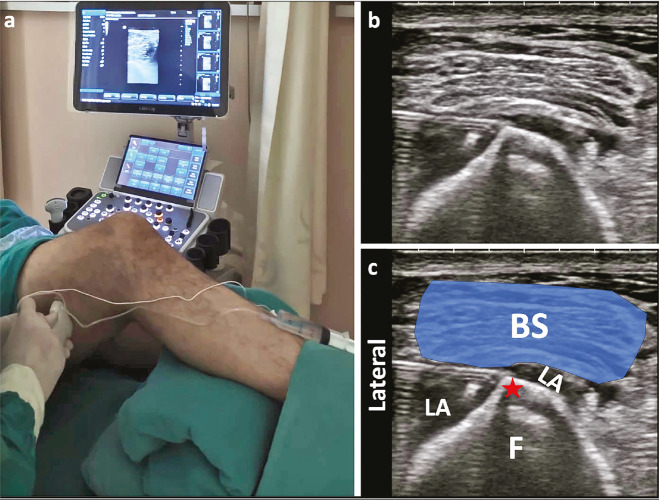
The biceps femoris short head block procedure: a. Patient and ultrasound probe positioning, b. Corresponding ultrasound image of the target area, c. Schematic illustration of the relevant anatomy, depicting the short head of the biceps femoris muscle (BS), the spread of local anaesthetic (LA), and the femoral shaft (F). The red star marks the lateral supracondylar line of the femur, the key anatomical landmark for needle guidance.
